# Draft Genome Sequence of a *bla*_NDM-1_- and *bla*_PME-1_-Harboring Pseudomonas aeruginosa Clinical Isolate from Pakistan

**DOI:** 10.1128/MRA.00107-19

**Published:** 2019-04-25

**Authors:** Sidra Irum, Robert F. Potter, Rubina Kamran, Zeeshan Mustafa, Meghan A. Wallace, Carey-Ann D. Burnham, Gautam Dantas, Saadia Andleeb

**Affiliations:** a Atta-Ur-Rahman School of Applied Biosciences (ASAB), National University of Sciences and Technology (NUST), Islamabad, Pakistan; b Pakistan Institute of Medical Sciences (PIMS), Islamabad, Pakistan; c The Edison Family Center for Genome Sciences and Systems Biology, Washington University in St. Louis School of Medicine, St. Louis, Missouri, USA; d Department of Pathology and Immunology, Washington University in St. Louis School of Medicine, St. Louis, Missouri, USA; e Department of Pediatrics, Washington University in St. Louis School of Medicine, St. Louis, Missouri, USA; f Department of Molecular Microbiology, Washington University in St. Louis School of Medicine, St. Louis, Missouri, USA; g Department of Biomedical Engineering, Washington University in St. Louis, St. Louis, Missouri, USA; Georgia Institute of Technology

## Abstract

We performed Illumina whole-genome sequencing on a carbapenem-resistant Pseudomonas aeruginosa strain isolated from a cystic fibrosis patient with chronic airway colonization. The draft genome comprises 6,770,411 bp, including the carbapenemase *bla*_NDM-1_ and the extended-spectrum beta-lactamase *bla*_PME-1_. This isolate harbors 3 prophages, 14 antibiotic resistance genes, and 257 virulence genes.

## Abstract (precis)

We performed Illumina whole-genome sequencing on a carbapenem-resistant Pseudomonas aeruginosa strain isolated from a cystic fibrosis patient with chronic airway colonization. The draft genome comprises 6,770,411 bp, including the carbapenemase *bla*_NDM-1_ and the extended-spectrum beta-lactamase *bla*_PME-1_.

## ANNOUNCEMENT

*Pseudomonas aeruginosa* is a Gram-negative opportunistic pathogen frequently involved in nosocomial infections (1, 2). We isolated P. aeruginosa strain PA-81 from tracheal secretions of a cystic fibrosis patient admitted to a tertiary care hospital in Pakistan. The sample was plated on blood agar (Oxoid, UK) followed by substreaking of morphologically distinct colonies on *Pseudomonas* cetrimide agar (Oxoid). The isolate was transferred to St. Louis, MO, for further characterization. Distinct colonies of the isolate were used for identification by Vitek MS matrix-assisted laser desorption ionization–time of flight mass spectrometry (MALDI-TOF MS) with the library v2.3.3 (bioMérieux, Durham, NC) using default settings. Because the strain was found to be resistant to all available classes of antibiotics, including cefepime, meropenem, piperacillin-tazobactam, ceftolozane-tazobactam, ceftazidime-avibactam, ciprofloxacin, aztreonam, and trimethoprim-sulfamethoxazole, this strain was selected for further analysis from among more than 200 P. aeruginosa isolates collected in 2016.

Genomic DNA was extracted from overnight growth on blood agar (Hardy Diagnostics, Santa Maria, CA) using the QIAamp BiOstic bacteremia DNA kit (Qiagen, Germantown, MD). Illumina sequencing libraries were prepared using the Nextera DNA flex library prep kit (Illumina, San Diego, CA) with 0.5 ng genomic DNA (3). Whole-genome sequencing was performed on an Illumina NextSeq 500 instrument to obtain 2 × 150-bp reads. Raw reads were processed and analyzed on the High Performance Computing Center cluster at Washington University in St. Louis School of Medicine, St. Louis, MO. Adapter sequences were trimmed using Trimmomatic v0.36 and decontaminated using DeconSeq v0.4.3 (4, 5). SPAdes v3.11.0 was used for *de novo* assembly of paired-end reads totaling 6,588,272 bp to produce an assembly with a mean genome coverage of 22× (6). The quality of the resulting assembly was evaluated using QUAST v4.5 (7). The assembly produced 331 contigs (largest contig, 580,364 bp) with an *N*_50_ value of 265,381 bp. PATRIC v3.5.20 was used for annotation, which determined a total genome length of 6,770,411 bp with 6,607 coding sequences (CDSs). The strain had a 66% GC content, 65 tRNA genes, 3 rRNA genes, and the 16S, 23S, and 5S loci (8).

The genome of strain PA-81 contained 614 unique genes that were absent from the genome of the PAO1 reference strain (NCBI Reference Sequence number NC_002516). The strain belongs to sequence type 357 (ST357) (https://cge.cbs.dtu.dk/services/MLST/) and serotype O11 (https://cge.cbs.dtu.dk/services/PAst/). Antibiotic resistance gene (ARG) annotation using the ResFinder and Comprehensive Antibiotic Resistance Database (CARD) servers (9, 10) revealed a number of ARGs against multiple classes of antibiotics, including beta-lactams (*bla*_OXA-10_, *bla*_OXA-50_, *bla*_PAO_, *bla*_Amp-C_, *bla*_NDM-1_, and *bla*_PME-1_), aminoglycosides [*aph(3′)-IIb*, *aph(3′)-VIa*, *ant(2ʺ)-Ia*, and *aadA1*], fosfomycin (*fosA*), trimethoprim (*dfrB2*), amphenicols (*catB7*), and sulfonamides (*sul1*). The presence of *bla*_NDM-1_ provides a likely mechanism of carbapenem resistance (11, 12). Further analysis using PlasmidFinder v1.3 did not reveal any known plasmid replicons (13). Three prophage regions of 21 kb, 12.6 kb, and 9.6 kb with identity scores of 90%, 85%, and 10%, respectively, were identified using PHAge Search Tool-Enhanced Release (PHASTER) (14). Default settings were used for all the employed tools and software.

To the best of our knowledge, this is the first draft genome sequence of a *bla*_NDM-1_- and *bla*_PME-1_-harboring P. aeruginosa strain from Pakistan. A more detailed analysis of this genome will be reported in a future publication.

### Data availability.

This whole-genome shotgun project has been deposited at DDBJ/ENA/GenBank under the accession number QXJN00000000 (version QXJN01000000) and SRA accession number SRR8510690.
